# Oxidized-LDL inhibits testosterone biosynthesis by affecting mitochondrial function and the p38 MAPK/COX-2 signaling pathway in Leydig cells

**DOI:** 10.1038/s41419-020-02751-z

**Published:** 2020-08-14

**Authors:** Jun Jing, Ning Ding, Dandan Wang, Xie Ge, Jinzhao Ma, Rujun Ma, Xuan Huang, Kadiliya Jueraitetibaike, Kuan Liang, Shuxian Wang, Siyuan Cao, Allan Zijian Zhao, Bing Yao

**Affiliations:** 1 Jinling Hospital Department Reproductive Medical Center, Clinical School of Medical College, Nanjing University, Nanjing, Jiangsu, China; 2 State Key Laboratory of Reproductive Medicine, Nanjing Medicine University, Nanjing, Jiangsu China; 3 The School of Biomedical and Pharmaceutical Sciences, Guangdong University of Technology, Guangzhou Guangdong, China

**Keywords:** Cell signalling, Endocrine reproductive disorders

## Abstract

Abnormal lipid/lipoprotein metabolism induced by obesity may affect spermatogenesis by inhibiting testosterone synthesis in Leydig cells. It is crucial to determine which components of lipoproteins inhibit testosterone synthesis. Circulating oxidized low-density lipoprotein (oxLDL), the oxidized form of LDL, has been reported to be an independent risk factor for decreased serum testosterone levels. However, whether oxLDL has a damaging effect on Leydig cell function and the detailed mechanisms have been rarely studied. This study first showed the specific localization of oxLDL and mitochondrial structural damage in testicular Leydig cells of high-fat diet-fed mice in vivo. We also found that oxLDL reduced the mitochondrial membrane potential (MMP) by disrupting electron transport chain and inhibited testosterone synthesis-related proteins and enzymes (StAR, P450scc, and 3β‑HSD), which ultimately led to mitochondrial dysfunction and decreased testosterone synthesis in Leydig cells. Further experiments demonstrated that oxLDL promoted lipid uptake and mitochondrial dysfunction by inducing CD36 transcription. Meanwhile, oxLDL facilitated COX2 expression through the p38 MAPK signaling pathway in Leydig cells. Blockade of COX-2 attenuated the oxLDL-induced decrease in StAR and P450scc. Our clinical results clarified that the increased serum oxLDL level was associated with a decline in circulating testosterone levels. Our findings amplify the damaging effects of oxLDL and provide the first evidence that oxLDL is a novel metabolic biomarker of male-acquired hypogonadism caused by abnormal lipid metabolism.

## Introduction

Leydig cells are steroidogenic cells present in the interstitial compartment of the testes and mainly contribute to testosterone synthesis and secretion^1^. Secreted testosterone diffuses into the seminiferous epithelium and signals through the androgen receptor in Sertoli cells^2^, supporting spermatogenesis processes such as the maintenance of the blood-testes barrier, meiosis, Sertoli-spermatid adhesion, and spermiation^3–5^. Low testosterone levels in humans have been related to developmental defects of the male reproductive system^6,7^ and disorders of male reproductive function, such as spermatogenesis failure, oligozoospermia, and male infertility^8^. Our previous study found that there are many important local regulatory mechanisms that accelerate testosterone synthesis in Leydig cells, including gonadotropin-releasing hormone (GnRH) and annexin A5^9–11^. However, the cellular and molecular mechanisms underlying the steroidogenic impairment of Leydig cells are not clear.

Increasing evidence from both epidemiological and clinical studies have indicated that a higher prevalence of metabolic syndrome, which is a constellation of low high-density lipoprotein cholesterol (HDL-C), and increased very-low-density lipoprotein (VLDL) and triglyceride (TG) levels, was along with lower testosterone^12^. A large number of animal studies have shown that a high-fat diet-induced dyslipidemia can affect the morphophysiology of Leydig cells and the subsequent downregulation of steroidogenic proteins^13,14^. Cholesterol homeostasis of patients, who suffer from hyperlipidemia and metabolic syndrome, is considered to be related to male fertility^15^. Thus, lipid metabolism disorder may affect spermatogenesis by inhibiting testosterone synthesis in Leydig cells. It has also been reported that the level of lipid/lipoprotein are negatively associated with plasma testosterone in male mice^16^. However, the implicit factors and precise mechanisms by which components of the lipoproteins affect Leydig cell function are still unknown.

Lipid peroxidation, especially low-density lipoprotein (LDL) oxidation, is considered a marker of oxidative stress and inflammation. Circulating oxidized low-density lipoprotein (oxLDL), the oxidative modified form of LDL, is associated with obesity, metabolic syndrome, and cardiovascular disease in adults^17–19^. Testosterone deficiency together with higher concentrations of oxLDL has been reported in epidemiological studies; however, there is currently no evidence for a causal relationship^20,21^. Oxidative damage and inflammatory factors induced by significantly high levels of oxLDL are involved in the dysfunction and apoptosis of vascular endothelial cells^22,23^. In the pathogenesis of male infertility, Leydig cells are vulnerable to oxidative stress and inflammatory damage, which may cause impaired secretion of testosterone. Previous studies have indicated that increased oxLDL plasma concentration may be one of the independent risk factors for spermatogenesis^24^. High serum oxLDL levels induced by a high-cholesterol diet in a rat model were shown to have a more destructive effect on the reproductive system and significantly decrease serum testosterone levels^24^. It has been suggested that oxLDL may play an important role in lipid metabolism disorders affecting testosterone synthesis in Leydig cells.

Therefore, the present study aimed to determine the effects of oxLDL on steroidogenesis in Leydig cells to reveal the mechanism by which oxLDL negatively modulates testosterone production. Our findings amplify the damaging effects of oxLDL, begin to reveal its molecular mode of action, and provide the first evidence that oxLDL is a negative regulator of male fertility.

## Results

### HFD decreased testosterone production and induced testicular damage in mice

**Fig. 1:**
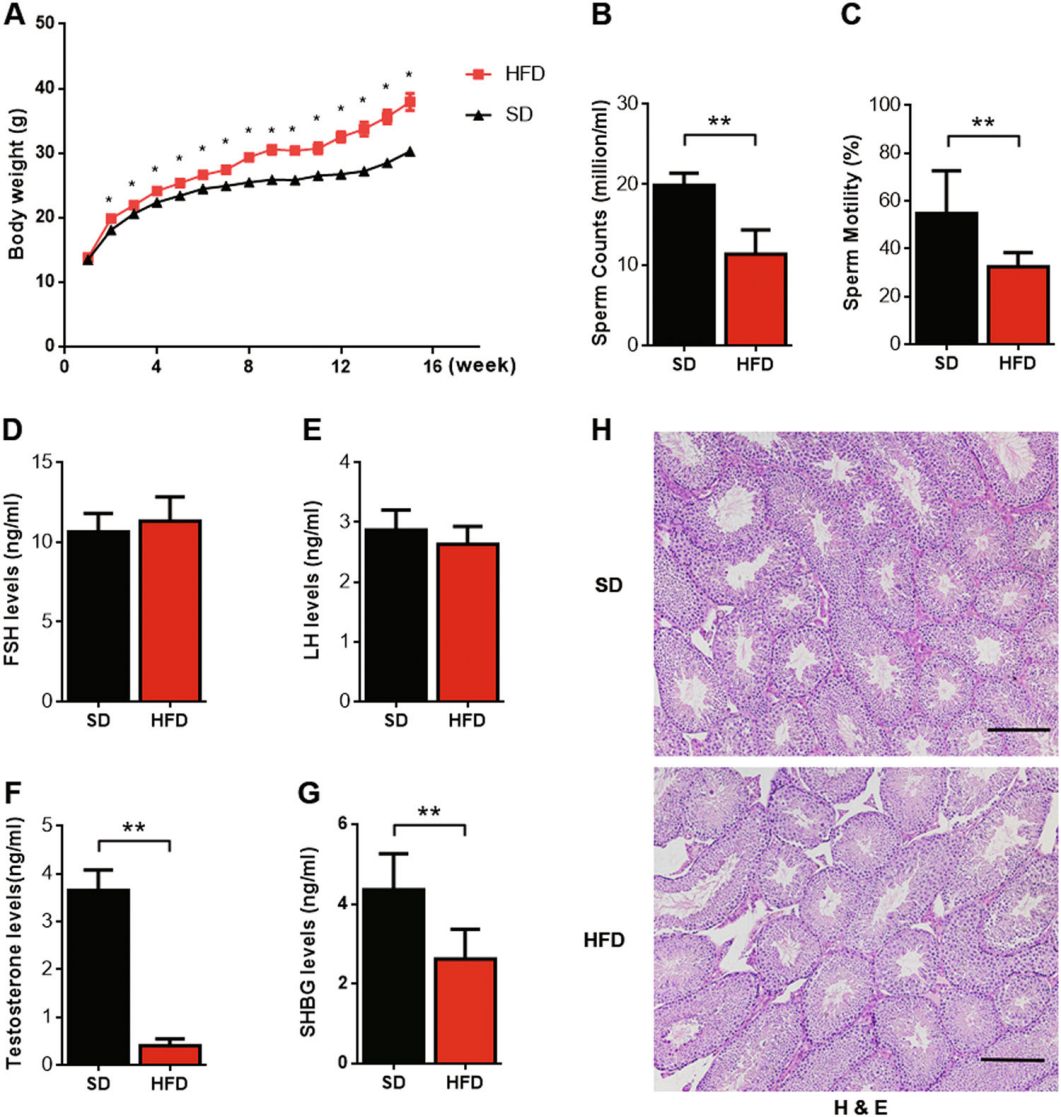
HFD-induced decreases in semen quality and testosterone production in mice. **a** The body weight of mice was measured every week, and the curve of body weight was plotted. **b** Sperm count was counted in a haemocytometer under a light microscope. **c** Sperm motility was analyzed by computer-assisted semen analysis. **d**–**g** Serum FSH, serum LH, serum testosterone, and serum SHBG levels were detected by ELISA. *P < 0.05, **P < 0.01, compared with SD-fed mice. **h** Hematoxylin and eosin (H&E) staining of histological sections of testicles in SD- and HFD-fed mice (*n* = 8 per group; scale bar, 200 μm).

To determine the function of oxLDL in Leydig cells, we first established a model by feeding a high-fat diet to 8-week-old B6 mice. The HFD significantly increased the body weight of the mice after 2 weeks, and the maximum body weight difference was achieved after 15 weeks (Fig. 1a). In contrast, sperm count and sperm motility were decreased significantly in the HFD-fed group (Fig. 1b, c). The serum testosterone and SHBG levels were decreased markedly in the HFD-fed group, while the serum FSH and LH levels were not significantly changed (Fig. 1d–g). Hematoxylin and eosin (H&E) staining was performed on histological sections of testes from mice that were provided a standard diet (SD) and HFD-fed mice (*n* = 8 per group; Fig. 1h). The testicular morphology of the SD-fed group was intact. Seminiferous tubules were arranged closely with narrow interstitial area, and the lumen of these tubules were full of spermatozoa. However, the testicular structures were abnormal in the HFD-fed group; the seminiferous tubules were separated from the loose interstitial area, and the number of germ cells in the tubules was significantly decreased.

### oxLDL levels were significantly elevated in the serum and testes of HFD-fed mice

**Fig. 2:**
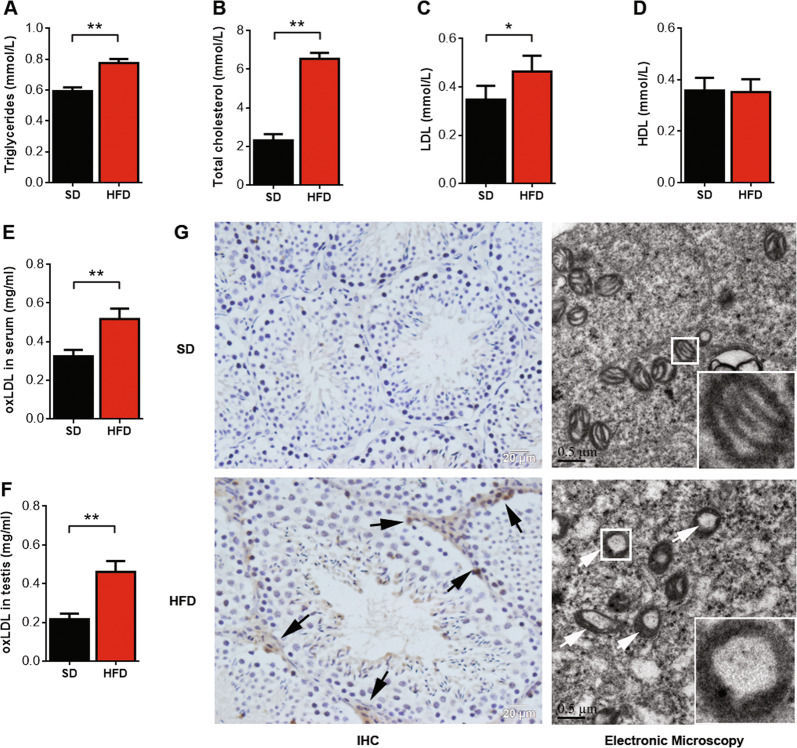
oxLDL level is significantly elevated in the serum and testes of HFD mice. Serum triglyceride (**a**), serum total cholesterol (**b**), serum LDL (**c**), and serum HDL (**d**) levels in mice were detected by a biochemical analyzer. Serum oxLDL (**e**) and testicular oxLDL (**f**) levels were measured by ELISA. *P < 0.05, **P <0.01, compared with SD-fed mice. (**g**) Immunohistochemical staining indicated that oxLDL accumulated in the testicles in SD- and HFD-fed mice, and the arrows show sites of positive expression (*n* = 8 per group; scale bar, 20 μm). Transmission electron microscopy showing the ultrastructural characteristics of mitochondria of Leydig cells in SD- and HFD-fed mice. The arrows show the absence of mitochondrial cristae (*n* = 8 per group; scale bar, 0.5 μm).

After successfully establishing the model, we measured the oxLDL levels in the mouse serum and testis, and the results indicated that oxLDL was significantly increased in HFD-fed mice (Fig. 2e, f). Immunohistochemical staining indicated that oxLDL was mainly accumulated in the Leydig cells of the HFD-fed mice. The ultrastructural characteristics of the mitochondria in the Leydig cells were established. The mitochondrial cristae in the SD-fed group were intact. However, mitochondrial inner membranes were disrupted, and cristae were absent in the HFD-fed group (Fig. 2g). Meanwhile, the serum levels of TG, TC, and LDL were increased significantly in the HFD-fed group, while the serum HDL levels were not significantly changed (Fig. 2a–d). Thus, the results indicate that oxLDL levels in Leydig cells may affect testosterone production.

### oxLDL-regulated testosterone production through the expression of StAR, P450scc, and 3β‑HSD in primary Leydig cells

**Fig. 3:**
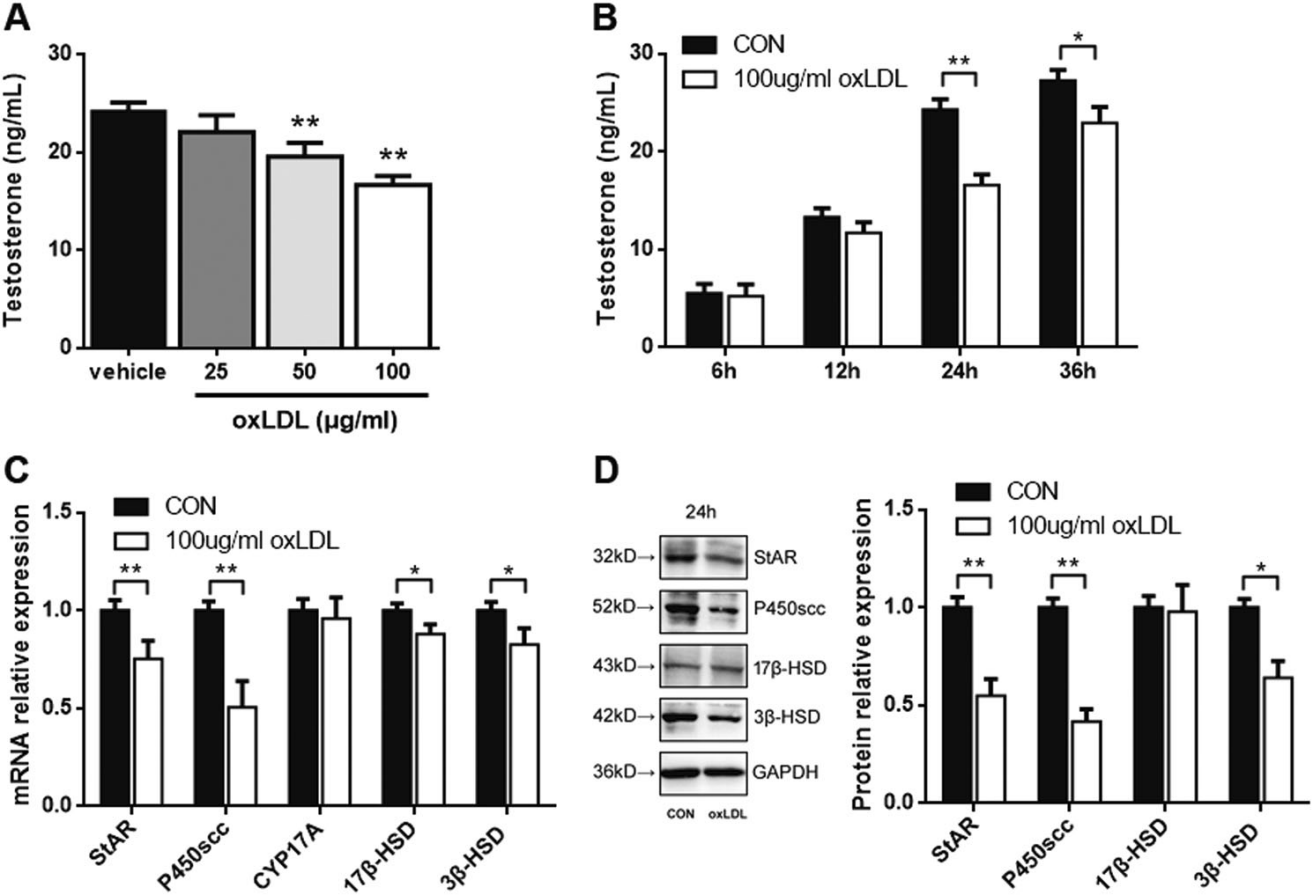
oxLDL inhibits testosterone production in primary Leydig cells. Cells were treated with various concentrations of oxLDL for 24 h (**a**) or with 100 μg/mL oxLDL for different time points (**b**). Then, the culture medium was collected and assayed for testosterone production. **c** Cells were treated with 100 μg/mL oxLDL for 24 h, and the mRNA expression of StAR, P450scc, CYP17A, 17β-HSD, and 3β-HSD was examined by qRT-PCR. **d** The protein expression of StAR, P450scc, 17β-HSD, and 3β-HSD was detected by western blotting. Quantitation of testosterone synthesis-related proteins and enzyme expression was determined by normalization to the internal control GAPDH. The vehicle with no oxLDL treatment was used as a control. The data are presented as the mean ± standard deviation of at least three independent experiments. *P < 0.05, **P < 0.01, compared with the control.

To determine whether oxLDL can inhibit testosterone production, primary Leydig cells were treated with different concentrations of oxLDL for 24 h. As shown in Fig. 3a, the level of testosterone was significantly decreased by 19 and 31% after treatment with 50 and 100 μg/mL oxLDL, respectively, for 24 h (*P* < 0.01), respectively. Then, oxLDL at 100 μg/mL was used to determine its temporal effect. Time‑dependent study indicated that the maximal effect of oxLDL on testosterone production was observed 24 h after treatment (*P* < 0.01; Fig. 3b). Therefore, a dose of 100 μg/mL and a time point of 24 h were used in the following experiments.

To evaluate the effect of oxLDL on the expression of testosterone synthesis-related proteins and enzymes, such as StAR, P450scc, CYP17A, 17β‑HSD, and 3β‑HSD, primary rat Leydig cells were treated with 100 μg/mL oxLDL for 24 h. The mRNA levels of these genes were examined by qRT‑PCR. As shown in Fig. 3c, compared with those in the control group, the mRNA levels of StAR, P450scc, 17β‑HSD, and 3β‑HSD were markedly decreased (*P* < 0.05), while CYP17A mRNA expression was not significantly affected (*P* > 0.05). Moreover, the expression of these proteins was examined by western blotting. 17β‑HSD protein expression was not affected after oxLDL treatment for 24 h, while the protein expression of StAR, P450scc, and 3β‑HSD was significantly decreased respectively (*P* < 0.05; Fig. 3d). Based on these findings, we suggest that oxLDL can inhibit the expression of StAR, P450scc, and 3β‑HSD, thus inhibiting testosterone production in Leydig cells.

### oxLDL disrupted mitochondrial function in primary Leydig cells

**Fig. 4:**
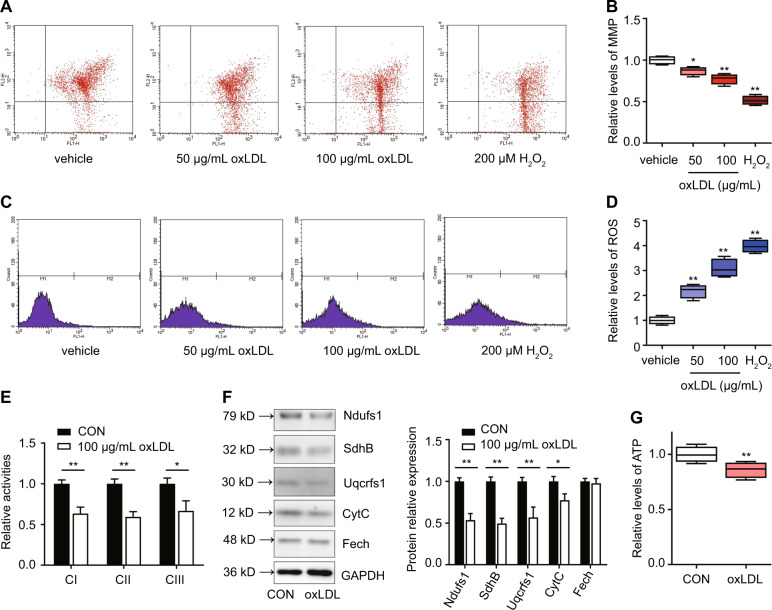
oxLDL inhibits mitochondrial function in primary Leydig cells. Cells were treated with various concentrations of oxLDL or 200 μM H_2_O_2_ for 24 h, and then the mitochondrial membrane potential (MMP) (**a**, **b**) and intracellular ROS (**c**, **d**) were assayed by flow cytometry. H_2_O_2_ as a positive control. **e** Activities of mitochondrial complexes I, II, and III (CI, CII, and CIII) were assessed by spectrophotometry. **f** The expression of respiratory chain proteins, including Ndufs1 (a subunit of CI), SdhB (a subunit of CII), Uqcrfs1 (a subunit of CIII), Fech (a matrix enzyme ferrochelatase), and CytC (an intermembrane space protein cytochrome C), was detected by western blotting. Quantitation of mitochondrial proteins expression was determined by normalization to the internal control GAPDH. **g** The level of Intracellular ATP were detected. The vehicle with no oxLDL treatment was used as a control. The data are presented as the mean ± standard deviation of at least three independent experiments. *P < 0.05, **P < 0.01, compared with the control.

Mitochondrial function is an important factor for guaranteeing testosterone synthesis in Leydig cells^13^. To determine whether oxLDL can affect mitochondrial function, primary Leydig cells were treated with different concentrations of oxLDL for 24 h. As shown in Fig. 4a, b, the mitochondrial membrane potential (MMP) was examined by flow cytometry with JC-1 staining. The MMP was significantly decreased after treatment with 50 μg/mL and 100 μg/mL oxLDL for 24 h (*P* < 0.05). Moreover, intracellular ROS were examined by flow cytometry with H_2_DCFDA staining. Intracellular ROS were significantly increased after treatment with 50 μg/mL and 100 μg/mL oxLDL for 24 h (*P* < 0.05; Fig. 4c, d). The activities of mitochondrial complexes I, II, and III were significantly decreased after 100 μg/mL oxLDL treatment (*P* < 0.05; Fig. 4e), which was consistent with the levels of the Fe–S-containing subunits Ndufs1 (of complex I), SdhB (of complex II), and Uqcrfs1 (of complex III; *P* < 0.01; Fig. 4f). In addition, other mitochondrial proteins, such as cytochrome C (CytC, an intermembrane protein) was also decreased (*P* < 0.05), while ferrochelatase (Fech, a matrix protein) was not significantly affected (Fig. 4f). The level of ATP was significantly decreased after treatment with 100 μg/mL oxLDL for 24 h (*P* < 0.05; Fig. 4g). Based on these findings, we demonstrate that oxLDL can inhibit electron transport chain, thus disrupting mitochondrial function in Leydig cells.

### oxLDL promoted lipid uptake and mitochondrial dysfunction by inducing CD36 transcription

**Fig. 5:**
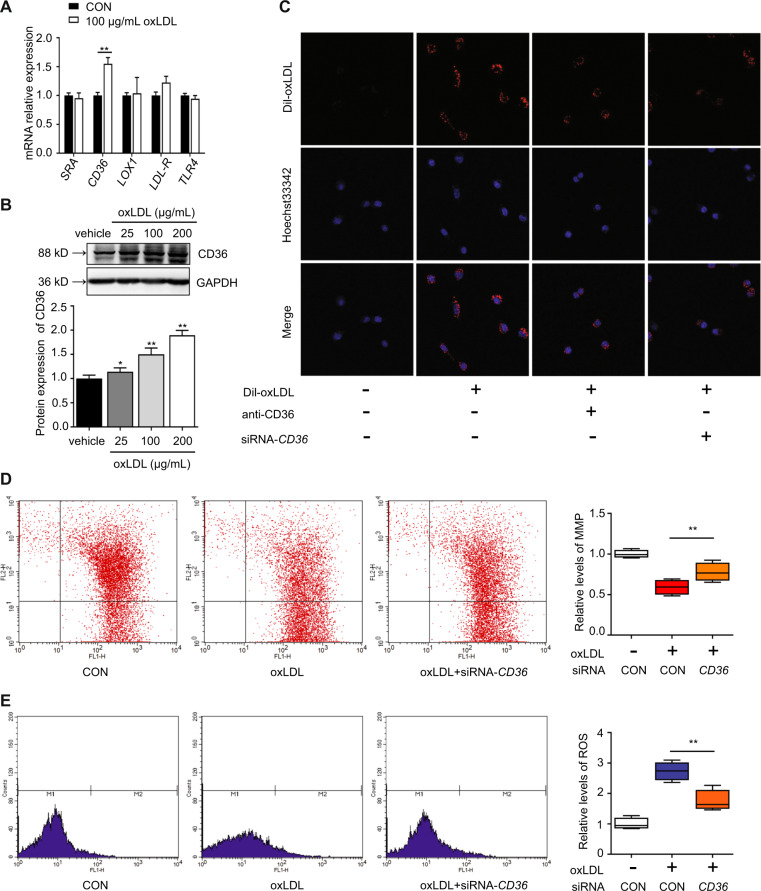
oxLDL promotes lipid uptake and mitochondrial dysfunction by inducing CD36 transcription in TM3 Leydig cells. **a** Cells were treated with 100 μg/mL oxLDL for 24 h, and the mRNA expression of the oxLDL-targeting scavenger receptors SRA, CD36, LOX1, LDL-R, and TLR4 was examined by qRT-PCR. **b** Cells were treated with various concentrations of oxLDL for 24 h, and CD36 protein expression was detected by western blotting. Quantitation of mRNA and protein expression was determined by normalization to the internal control GAPDH. No oxLDL treatment was used as a control. **c** Cells were preincubated with a CD36-specific antibody for 1 h or transfected with negative control (NC) or siRNA-CD36 for 24 h. Then, the cells were incubated with Dil-oxLDL for 30 min. Lipid uptake was evaluated by the fluorescence intensity of Dil-oxLDL. The cells were transfected with siRNA-CD36 or NC and then with blank control or 100 μg/mL oxLDL for 24 h. The mitochondrial membrane potential (MMP) (**d**) and intracellular ROS (**e**) were assayed by flow cytometry. The data are presented as the mean ± standard deviation of at least three independent experiments. *P < 0.05, **P < 0.01, compared with the control.

To understand which scavenger receptors mainly transport oxLDL into Leydig cells, changes in the oxLDL-targeting scavenger receptors SRA, CD36, LOX1, LDL-R, and TLR4 in TM3 Leydig cells were detected after treatment with 100 μg/mL oxLDL for 24 h. The CD36 mRNA level was significantly increased (*P* < 0.01), and the LDL-R mRNA level was also increased but not significantly (Fig. 5a). The CD36 protein levels were significantly increased (*P* < 0.05) in response to oxLDL at 25, 50, and 100 μg/mL, respectively (Fig. 5b). To examine the silencing effect of siRNAs on CD36 expression, we transfected 3 siRNA duplexes and scrambled siRNA into TM3 Leydig cells. We found that siRNA1 was the best among the three duplexes, and it was used in the following experiments (Fig. S1A–C). As shown in Fig. 5c, blocking or knocking down the cell membrane receptor CD36 partially decreased oxLDL uptake by Leydig cells, suggesting that oxLDL might be transported into Leydig cells through the induction of increased CD36 expression. As shown in Fig. 5d, e, oxLDL inhibition of mitochondrial function could be rescued by CD36 knockdown, which enhanced the MMP and reduced intracellular ROS levels. These results demonstrate that oxLDL could promote lipid uptake and mitochondrial dysfunction of Leydig cells by inducing CD36 transcription.

### COX-2 expression regulated by oxLDL was involved in the p38 MAPK signaling pathway

**Fig. 6:**
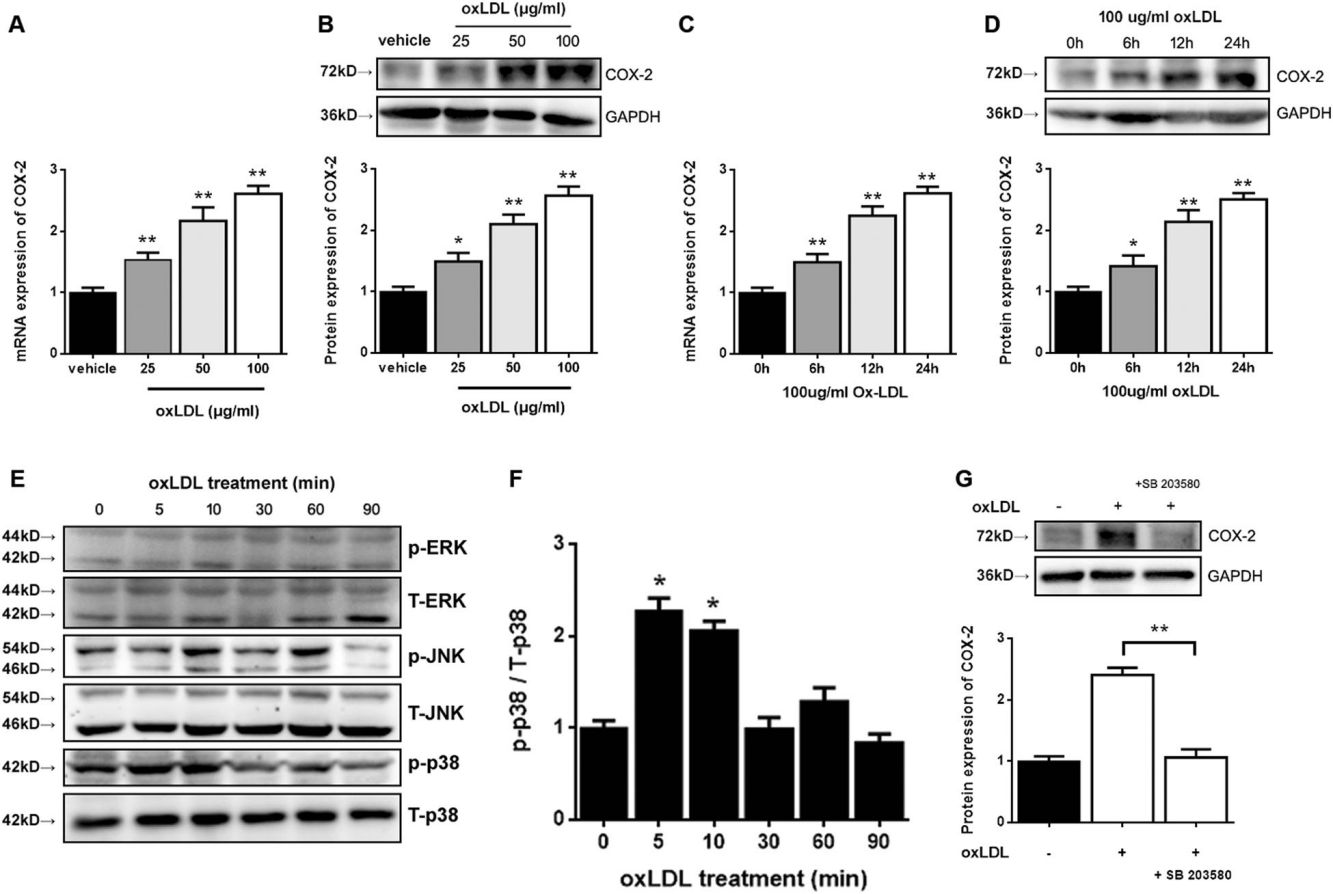
oxLDL-induced COX-2 expression is mediated by the p38 MAPK pathway in TM3 Leydig cells. Cells were treated with various concentrations of oxLDL for 24 h (**a**, **b**) or with 100 μg/mL oxLDL for different time points (**c**, **d**), and the mRNA and protein expression of COX-2 was detected by qRT-PCR and western blotting, respectively. The vehicle with no oxLDL treatment was used as a control. *P < 0.05, **P < 0.01, compared with the control. **e** After incubation with 100 μg/mL oxLDL for different time points, the levels of phosphorylated MAPK (p-ERK, p-JNK, and p-p38) and total MAPK (T-ERK, T-JNK, and T-p38) were detected by western blotting. **f** The ratio of p-p38 to T-p38 was calculated. The p-p38 level in TM3 cells without oxLDL treatment (0 min) was used as a control (*P < 0.05). **g** Cells were pretreated with 0.5 μM SB203580 (a p38 inhibitor) for 1 h and then with 100 μg/mL oxLDL for 24 h. The protein expression of COX-2 was detected by western blotting and compared to that observed with oxLDL treatment alone (**P < 0.01). The data are presented as the mean ± standard deviation of at least three independent experiments.

To determine the mechanism that underlies the regulation of testosterone production by oxLDL, we studied COX-2, which is overexpressed in aged Leydig cells. TM3 Leydig cells were treated with oxLDL either at different concentrations or for different durations, and COX-2 expression increased at both the mRNA and protein levels. oxLDL treatment at concentrations of 25, 50, and 100 μg/mL for 24 h significantly increased COX-2 mRNA expression (*P* < 0.05), respectively compared to baseline(Fig. 6a). COX-2 protein levels significantly increased (*P* < 0.05) in response to oxLDL at 25, 50, and 100 μg/mL, respectively (Fig. 6b). Treatment with 100 μg/mL oxLDL significantly induced COX-2 mRNA expression after 6, 12, and 24 h, respectively (*P* < 0.05; Fig. 6c). The protein levels also increased steadily 6-24 h after the administration of oxLDL. COX-2 protein levels were significantly increased (*P* < 0.05) after 6, 12, and 24 h, respectively (Fig. 6d).

To investigate whether oxLDL activates any of the MAPK signaling pathways in TM3 Leydig cells, we measured the phosphorylation levels of ERK1/2, JNK, and p38 MAPK by western blot analysis using antibodies that specifically recognize the phosphorylated forms of these MAPK proteins. When cells were treated with oxLDL at a concentration of 100 μg/mL for 90 min, the levels of p38 phosphorylation rapidly increased as early as 5 min after administration and remained elevated compared to baseline after 10 min (*P* < 0.05; Fig. 6f). However, the levels of unphosphorylated total p38 did not change after treatment, and the levels of ERK1/2 and JNK MAPK phosphorylation were not altered by oxLDL treatment (Fig. 6e). Pretreatment with SB203580 at a concentration of 0.5 μM for 1 h completely abolished the effect of oxLDL on p38 MAPK activation and repressed the increase in COX-2 expression at the protein level (*P* < 0.05; Fig. 6g). Together, these results demonstrate that oxLDL can facilitate COX-2 expression through the p38 MAPK signaling pathway.

### Blockade of COX-2 attenuated the oxLDL-induced decrease in StAR and P450scc

**Fig. 7:**
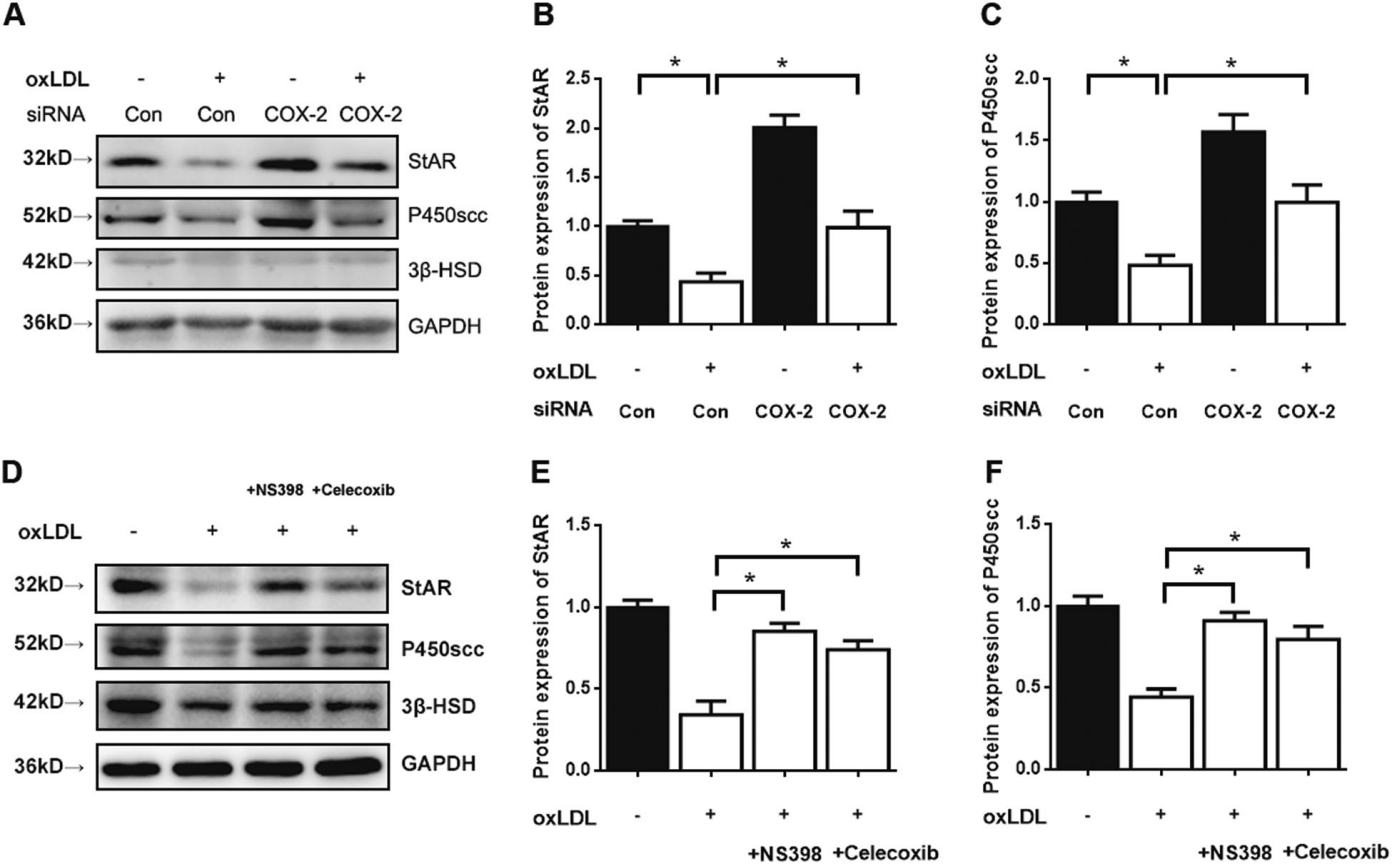
Blockade of COX-2 inhibits the downregulation of StAR and P450scc by oxLDL in TM3 Leydig cells. **a**–**c** The cells were transfected with siRNA-COX-2 or scrambled siRNA (negative control) and then with blank control or 100 μg/mL oxLDL for 24 h. **d**–**f** The cells were treated with COX-2 inhibitors NS398 (10 μM) or Celecoxib (25 μM) for 1 h and then with blank control or 100 μg/mL oxLDL for 24 h. The protein expression of StAR, P450scc, and 3β-HSD was detected by western blotting. *P < 0.05, compared with oxLDL treatment alone. The data are presented as the mean ± standard deviation of at least three independent experiments.

To examine the silencing effect of siRNAs on COX-2 expression, we transfected three siRNA duplexes and scrambled siRNA into TM3 Leydig cells. We found that siRNA3 was the best among the three duplexes, and it was used in the following experiments (Fig. S1d–f). To further explore the role of COX-2 in the oxLDL-mediated inhibition of Leydig cell testosterone production, we employed siRNA to assess the effect of COX-2 depletion on the oxLDL-induced decrease in StAR, P450scc, and 3β‑HSD protein expression. Compared to the control, the administration of 100 μg/mL oxLDL for 24 h caused a significant decrease in the protein expression of StAR, P450scc, and 3β‑HSD (*P* < 0.05). However, the decreases in StAR and P450scc but not 3β‑HSD induced by oxLDL was significantly attenuated by transfection with siRNA-COX-2 (*P* < 0.05; Fig. 7a–c) compared with scrambled siRNA. We have also used the chemical inhibitors of COX-2 (10 μM NS398 or 25 μM Celecoxib for 1 h pretreatment) to detect the testosterone synthesis-related proteins and enzymes. The protein levels of StAR and P450scc but not 3β‑HSD were dramatically rescued with both of the inhibitors treatment (*P* < 0.05; Fig. 7d–f) compared with oxLDL treatment alone. The results were similar with that of COX-2 knockdown. Thus, the results suggest that the involvement of COX-2 in the oxLDL-mediated inhibition of Leydig cell testosterone production.

### Negative correlation between oxLDL and testosterone levels in human serum

**Fig. 8:**
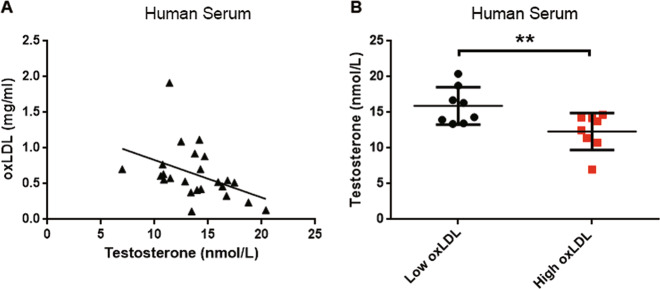
Negative correlation between oxLDL and testosterone in human serum. **a** Analysis of the correlation between oxLDL levels and testosterone levels in human male serum (*n* = 24). *R* = −0.510. **b** The testosterone levels in human male serum from the low oxLDL (<0.5 mg/ml) group and the high oxLDL (>0.7 mg/ml) group (*n* = 8 per group) were determined by a chemiluminescence assay. The data are presented as the mean ± standard deviation. **P < 0.01, compared with the low oxLDL group.

To investigate the relationship between oxLDL and testosterone in human serum, we measured oxLDL, lipid, and sex hormone levels in human serum (from male donors aged 24–41 years). The statistical analysis revealed that the oxLDL level was significantly correlated with the serum levels of TG (*r* = 0.669, *P* < 0.001), TC (*r* = 0.497, *P* = 0.014), and LDL (*r* = 0.454, *P* = 0.026), but not HDL (Table 1). The results also showed that the oxLDL level was negatively correlated with testosterone levels in human male serum (*r* = −0.510, *P* = 0.011; Fig. 8a), and the testosterone levels in the high oxLDL (>0.7 mg/ml) group were significantly lower than those in the low oxLDL (<0.5 mg/ml) group (*P* < 0.01; Fig. 8b). However, the results showed that the serum oxLDL levels were not correlated with sperm characteristics (*P* > 0.05; Table S1). The above results further suggest that increased serum oxLDL levels may be related to the decline of Leydig cell function in human males.

**Table 1:** Spearman’s correlations between the oxLDL level and age, the serum sex hormone level, and the serum lipid level.

	Age	Testo	FSH	LH	E2	TC	TG	LDL	HDL
oxLDL	0.349 (0.094)	−0.510 (**0.011**)	0.031 (0.885)	−0.146 (0.496)	0.207 (0.331)	0.497 (**0.014**)	0.669 (**0.000**)	0.454 (**0.026**)	−0.257 (0.225)

Data are presented as a correlation coefficient (*P*).

*oxLDL* oxidized low-density lipoprotein, *Testo* testosterone, *FSH* follicle-stimulating hormone, *LH* luteinizing hormone, *E2* estradiol, *TC* total cholesterol, *TG* triglyceride, *LDL* low-density lipoprotein, *HDL* high-density lipoprotein.

The bold values in brackets represents the *P* value.

## Discussion

Our previous study illuminated that abnormal lipid metabolism in the reproductive system has a direct impact on sperm function^25–27^. Many recent studies have suggested that a high-fat diet induces local regulatory mechanisms that control the inhibition of steroidogenesis by Leydig cells^28^. This study revealed the importance of CD36 in mediating the uptake of oxLDL in Leydig cells and that the accumulation of oxLDL in the interstitial compartment of the testes inhibits the mitochondrial function of Leydig cells and testosterone synthesis-related proteins and enzymes through the p38 MAPK/COX-2 signaling pathway, thereby causing a decrease in testosterone synthesis (Fig. S2).

A previous study found that diet-induced obesity increases Sertoli cell apoptosis and lipotoxicity, accompanied with elevated free fatty acid levels in the testes^29,30^. Meanwhile, the factors and mechanisms by which obesity interferes with spermatogenesis have been shown to be dysfunction of Leydig cells and a decrease in testicular testosterone synthesis^31^. In the present study, mice fed with HFD were used as an animal model of hyperlipidemia. We also found that a high-fat diet caused a decline in the fertility of male mice, including decreased sperm count, sperm motility, and testosterone levels. At the same time, we found that serum and testicular oxLDL levels were significantly elevated. In our study, we observed for the first time the specific localization of oxLDL and mitochondrial structure damage in testicular Leydig cells of high-fat diet-fed mice in vivo. Collectively, these findings indicate a substantial negative impact of oxLDL on Leydig cell function and male reproductive function (Figs. 1 and 2). However, the animal model in our study have some drawbacks to study the function of oxLDL in testicular Leydig cells. HFD-fed mice developed obesity with increasing in leptin secretion, which has been shown to negatively influence Leydig cell function^32–34^. Furthermore, HFD-fed mice developed insulin resistance, which is associated with a decrease in Leydig function^35^. There are also other possible influences on Leydig cell function by other hormones secreted or not during HFD-diet (Ghrelin for example) but their role in Leydig cell function modulation has not been clearly demonstrated yet^36^. In addition, the latest data have illustrated that decreased testosterone levels in an atherosclerosis model might result from both the rarefication of the capillary network in the testes and decreased Leydig cell number and size^37^. Since oxLDL is an important biomarker of cardiovascular diseases, we will research the function of oxLDL in testicular Leydig cells in an atherosclerosis model.

Mitochondrial structure and function are important factors that guarantee testosterone synthesis in Leydig cells^13^. The steroid hormone synthesis acute regulatory protein (StAR), which is a rate-limiting step in steroid hormone synthesis, is located in the mitochondrial membrane^38^. Cholesterol transported into the mitochondrial matrix is further converted into pregnenolone by cytochrome P450scc, which is also located in the mitochondrial membrane, then converted into testosterone by 3β-HSD, CYP17A, and 17β-HSD in the smooth endoplasmic reticulum^39^. Papers have also found that Leydig cell function could be inhibited by increasing intracellular reactive oxygen species (ROS) and disrupting mitochondrial membrane permeability^40,41^. In our study, we determined that oxLDL inhibits testosterone biosynthesis not only by inhibiting the expression of testosterone synthesis-related proteins and enzymes, but also by destroying mitochondrial function via disrupting electron transport chain (Figs. 3 and 4), which is consistent with previous findings. Mitochondrial dysfunction aggravated the inhibition of oxLDL in testosterone biosynthesis, but whether it is direct reason for testosterone decline still need to be explored.

Several types of scavenger receptors (SRs), such as SR-AI/II, low-density lipoprotein receptor (LDL-R), CD36, lectin-like oxidized low-density lipoprotein receptor 1 (LOX1), and toll-like receptors (TLRs), can promote the internalization of oxLDL^42,43^. Previous studies have reported that oxLDL genetically induces the expression of LOX-1 in Chinese hamster ovary cells^44^. Our experiments found that oxLDL treatment increased the mRNA and protein expression of the scavenger receptor CD36 in TM3 Leydig cells. We also observed that both the blockade and knockdown of the cell membrane receptor CD36 partly decreased oxLDL uptake and mitochondrial dysfunction (Fig. 5). These findings clarify that oxLDL might promote lipid uptake and mitochondrial dysfunction mainly by increasing CD36 transcription in Leydig cells.

Cyclooxygenase-2 (COX-2), an isoform of cyclooxygenase, is overexpressed in aged Leydig cells^45^. The expression of COX-2 can be induced by oxLDL in macrophages^46,47^. Previous studies have shown that oxidative stress can induce COX-2 expression through p38/MAPK signaling^48^. MAPKs have been associated with disturbances in spermatogenesis and the dysfunction of germ and Sertoli cells, thus bringing about reduced semen quality and male reproductive dysfunction^49^. OxLDL affected directly inflammatory response, oxidative stress, cell permeability, and other cellular functions through MAPK signaling pathways^50^. P38/MAPK can induce the activation of the apoptosis pathway in the mitochondria of Leydig cells^51,52^. In this study, we figured out that oxLDL increased COX-2 expression through p38/MAPK signaling in TM3 Leydig cells (Fig. 6a–d). OxLDL caused a rapid and transient increase in the level of phosphorylated p38, but ERK1/2 and JNK/MAPK were not significantly activated in TM3 Leydig cells. Therefore, p38 may be the only MAP kinase that mediates the oxLDL-induced expression of COX-2 in these cells. In addition, p38 inhibitor blocked COX-2 induction (Fig. 6e–g). These results indicate an essential role for p38 MAPK signaling in the regulation of COX-2 and the mediation of oxLDL-inhibited steroidogenesis in Leydig cells. However, whether other oxLDL-regulated factors or signaling pathways further mediate the regulation of testosterone production requires additional investigation. For example, the NF-κb/COX-2 signaling pathway is also involved in the inhibition of testosterone synthesis in aging Leydig cells^53^. Next, we verified that blockade of COX-2 attenuated the oxLDL-induced downregulation of StAR and P450scc in TM3 Leydig cells (Fig. 7). Earlier studies have reported that COX-2-generated two arachidonic acid (AA) metabolites, 5-HPETE and 5-HETE, could inhibit StAR gene expression by inducing transcriptional repressors in aging Leydig cells^54^. COX2-dependent inhibition of P450scc expression is not clear currently. Therefore, the mechanism responsible for the COX2-dependent inhibition of StAR and P450scc expression with oxLDL treatment needs to be further verified.

Reduced serum testosterone is associated with a number of metabolic changes, including decreased lean body mass, increased adiposity, and cardiovascular disorders^55^. Our clinical results clarified that the increased serum oxLDL level may be related to the decline of circulating testosterone levels, reflecting Leydig cell function, in human males (Fig. 8). However, serum oxLDL levels were not associated with sperm characteristics. This may be because serum oxLDL does not penetrate the blood-testicular barrier and ultimately affects spermatogenesis. This suggests that oxLDL may be used as a novel metabolic biomarker, indicating that men are diagnosed with acquired hypogonadism due to abnormal lipid metabolism.

This study reveals that oxLDL is a negative regulator of male fertility. This interaction is mediated through oxLDL, which binds to the CD36 receptor, which is present on Leydig cells in the testes, and damages testosterone biosynthesis through affecting mitochondrial function and the p38 MAPK/COX-2 signaling pathway.

## Materials and methods

All chemicals and culture media were purchased from Sigma-Aldrich; Merck KGaA (Darmstadt, Germany) unless stated otherwise. C57BL/6J mice and Sprague-Dawley rats were used in this study. All animal experiments were approved by the Animal Care and Use Committee of Jinling Hospital and were performed in accordance with institutional guidelines.

### Antibodies

Rabbit polyclonal anti-oxidized LDL (cat. no. ab14519), rabbit polyclonal anti-COX-2 (cat. no. ab15191), rabbit monoclonal anti-3β-HSD (cat. no. ab150384), mouse monoclonal anti-phospho-p38 (cat. no. ab45381), rabbit monoclonal anti-p38 (cat. no. ab170099), rabbit monoclonal anti-phospho-JNK (cat. no. ab124956), and rabbit monoclonal anti-JNK (cat. no. ab208035) were purchased from Abcam (Cambridge, MA, USA); rabbit monoclonal anti-phospho-ERK1/2 (cat. no. #4370) and rabbit monoclonal anti-ERK1/2 (cat. no. #4695) were purchased from Cell Signaling Technology (Beverly, MA, USA); rabbit polyclonal anti-STAR (cat. no. 12225-1-AP), rabbit polyclonal anti-p450scc (cat. no. 13363-1-AP), rabbit polyclonal anti-CD36 (cat. no. 18836-1-AP), rabbit polyclonal anti-Ndufs1 (cat. no. 12444-1-AP), rabbit polyclonal anti-SdhB (cat. no. 10620-1-AP), rabbit polyclonal anti-Uqcrfs1 (cat. no. 18443-1-AP), rabbit polyclonal anti- Fech (cat. no. 14466-1-AP), and rabbit polyclonal anti-CytC(cat. no. 10993-1-AP) were purchased from ProteinTech Group (Chicago, IL, USA); rabbit polyclonal anti-17β-HSD (cat. no. sc-32872) was purchased from Santa Cruz Biotechnology (Eugene, OR, USA); mouse monoclonal GAPDH (cat. no. KC-5G5) was purchased from KangChen Biotech (Shanghai, China); and horseradish peroxidase (HRP)-conjugated goat anti-rabbit IgG (H + L) secondary antibody and HRP-conjugated goat anti-mouse IgG (H + L) secondary antibody were purchased from Thermo Fisher Scientific, Inc. (Waltham, MA, USA).

### Animals and diet

Male C57BL/6J mice were bred in our laboratory. The temperature and humidity of the animal room were maintained at 22 ± 2 °C and 53 ± 2%, respectively. A constant 12-h light:12-h dark cycle was set. Food and water were freely available. In all, 8-week-old mice were divided into two groups (*n* = 8 per group) by random number generating method as follows: control mice, which were fed a standard diet (SD; 4.3% fat, 19.2% protein, and 67.3% carbohydrate by energy; Research Diet, New Brunswick, USA), whereas HFD-fed mice were fed a high-fat diet (HFD; 34.9% fat, 26.2% protein, and 26.3% carbohydrate by energy) for 16 weeks. Spermatozoa from the left cauda epididymis tissue were released into 0.5 ml of HTF medium containing 0.5% bovine serum albumin and incubated for 5 min at 37 °C. Sperm motility were determined immediately after the sperm collection, using the standard method on a haemocytometer under a light microscope. The number of motile spermatozoa was calculated per unit area and expressed as percentage of sperm motility. Sperm count was also determined using a haemocytometer, and the results were expressed as million ml^−1^ of suspension.

### Histopathological analyses and immunohistochemistry

Specimens of the testes were fixed in 4% paraformaldehyde. Following routine pathological procedures, section slides were prepared. Subsequently, the slides were stained with hematoxylin-eosin (H&E). For DAB staining and immunohistochemical analysis, the slides were treated with 3% hydrogen peroxide (H_2_O_2_) solution to block endogenous peroxidase activity. Nonspecific binding sites were blocked with 1% BSA in PBS for 1 h at room temperature and subsequently incubated with rabbit polyclonal anti-oxLDL (1:200) overnight at 4 °C. The slides were then washed and incubated with an anti-rabbit IgG secondary antibody. Protein expression was visualized with DAB (Dako Cytomation, Carpinteria, CA) staining. The reaction was stopped with distilled water, and the slides were stained with hematoxylin and dehydrated before mounting. The images were digitized by fluorescence microscopy on an IX73 microscope (Olympus Corporation, Shinjuku, Tokyo, Japan).

### Transmission electron microscopy

Specimens of the testes were fixed in culture dishes for 30 min in PBS containing 0.5% glutaraldehyde. After immobilization in 2% agarose, the samples were incubated for 2 h in an aqueous solution of 1% OsO_4_ containing 1.5% hexacyanoferrate (II), washed in water, and stored in 1% aqueous uranyl acetate overnight at 4 °C. After washes in water and dehydration in acetone, the samples were embedded in Epon. Ultrathin sections (60 nm) were mounted on formvar-coated copper grids, poststained with uranyl acetate and lead citrate, and observed using a transmission electron microscope (JEM-1011, JEOL, Japan). Images were recorded using a side-mounted CCD camera.

### Detection of reproductive hormone and lipid levels

The reproductive hormone and lipid levels in the serum and/or testes were measured according to the manufacturers’ instructions. Briefly, blood samples were centrifuged at 3000 × *g* for 5 min to isolate the serum for the detection of reproductive hormone and lipid levels. Meanwhile, 0.1 g of testis tissue was homogenized with 500 µl of ice-cold radioimmunoprecipitation assay buffer (RIPA, Sigma, USA) and centrifuged at 4 °C at 12,000 rpm for 15 min. The supernatant was then transferred to a new EP tube for oxLDL measurement. Triglyceride, total cholesterol, HDL, and LDL serum concentrations were measured by a Hitachi 7600-210 automatic biochemical analyzer. Follicle-stimulating hormone (FSH), luteinizing hormone (LH), testosterone, sex hormone binding globulin (SHBG), and oxLDL levels in all samples were analyzed using an available ELISA kit (Cloud-Clone Corp, Wuhan, China) according to the manufacturer’s instructions.

### Primary Leydig cell isolation and culture

Male Sprague-Dawley rats (9–10 weeks old) were bred in our laboratory. Leydig cells were prepared from immature rat testes by collagenase treatment as described previously^9^. Briefly, decapsulated testes were incubated with collagenase (0.25 mg/mL) for 20 min at 37 °C. The crude interstitial cells were collected by centrifugation at 1000 × *g* for 10 min and then washed twice with HBSS containing 0.1% (w/v) BSA. To obtain pure Leydig cells, the crude cell suspension was loaded onto a discontinuous Percoll gradient (20, 40, 60, and 90% Percoll in HBSS) and subsequently centrifuged at 800 × *g* for 20 min. The fractions enriched in Leydig cells were obtained and centrifuged in a continuous, self-generating density gradient starting with 60% Percoll at 20,000 × *g* for 30 min at 4 °C. The total number of cells and the percentage of 3β-HSD-positive cells with this Leydig cell preparation were determined. The purity of the Leydig cells was 85–90%. The cell viability, as assessed by Trypan blue exclusion, was >90%. The purified Leydig cells were washed twice with DMEM-F/12 and resuspended in DMEM-F/12 supplemented with 15 mmol/L HEPES (pH 7.4), 1 mg/mL BSA, 365 mg/L glutamine, 100 IU/mL penicillin, and 100 mg/mL streptomycin. For culturing, 2 mL cell suspension containing 10^6^ cells/mL was placed into each well of a 6-well plate (Costar, NY, USA) and incubated at 34 °C in a humidified atmosphere of 5% CO_2_–95% air.

### TM3 cell culture and treatment

A mouse Leydig cell line (TM3) was purchased from Procell Life Science & Technology Co. Ltd. (Wu Hai, China). The TM3 cells were cultured in DMEM/F12 supplemented with 5% horse serum and 2.5% fetal bovine serum. The cells were grown at 37 °C in an atmosphere of 5% CO_2_ in air.

OxLDL was purchased from Yiyuan, Guangzhou, China (by 5 μM Cu_2_SO_4_-induced LDL oxidation in PBS at 37 °C for 20 h, Malondialdehyde 19.2 nmoles of MDA/mg Protein). Twenty-four hours after plating, the medium of all of the cells was changed to fresh medium, and the cells were subjected to the experimental conditions described below. The primary and TM3 Leydig cells were incubated with fresh medium containing increasing concentrations of oxLDL (0, 25, 50, and 100 μg/mL) or with 100 μg/mL oxLDL for different times (0, 6, 12, 24 and 36 h). The culture medium was collected and stored at −20 °C for testosterone measurement, and the cells were harvested for flow cytometry, and RNA and protein extraction. In another experiment, TM3 cells were treated with 100 μg/mL oxLDL and harvested at different time points (0, 5, 10, 30, 60, and 90 min) for western blot analysis to determine the levels of phosphorylated MAPK (ERK1/2, JNK, and p38). In parallel, some cells were preincubated with the p38 MAPK inhibitor SB203580 (0.5 μM; Selleck), COX-2 inhibitors NS398 (10 μM; Selleck), or Celecoxib (25 μM; Selleck) for 1 h and then with 100 μg/mL oxLDL for another 24 h. The cells were harvested for protein extraction.

### Transfection of siRNAs

A set of mouse CD36-specific siRNA (siRNA-CD36), a set of mouse COX-2-specific siRNA (siRNA-COX-2) and nonspecific siRNA (scrambled siRNA; negative control) were designed and synthesized by GenePharma (Shanghai, China). To knockdown the expression of CD36 and COX-2, the cells were transfected with siRNA-CD36 or siRNA-COX-2 for 6 h before the indicated treatments. Transfection of the siRNAs was performed using Lipofectamine 3000 reagent (cat. no. L3000015; Invitrogen, Thermo Fisher Scientific, Inc.) according to the manufacturer’s instructions. The siRNA-CD36 and siRNA-COX-2 sequences are shown in Table S2.

### oxLDL uptake analysis

TM3 cells were preincubated with a sufficient amount of CD36-specific antibody (1:100) to block the cell membrane receptor CD36 for 1 h at 37 °C. Meanwhile, TM3 cells were transfected with negative control (NC) or siRNA-CD36 for 24 h, and then the cells were treated with Dil-oxLDL (10 ng/ml; red; Yiyuan, Guangzhou, China) for 30 min. Hoechst 33342 (blue) was used for chromosome staining. Next, the cells were washed twice with ice-cold PBS and observed under a laser scanning confocal microscope (LSM 710, Zeiss, Germany) equipped with a ×40 objective.

### Intracellular ROS and mitochondrial membrane potential by flow cytometry assays

The MMP was measured by JC-1 fluorescent staining. The JC-1 detection kit was purchased from KeyGEN BioTECH (Nanjing, China). Briefly, 3 × 10^6^ cells were suspended in 1 mL of PBS containing 10 mg/mL JC-1 and incubated for 10 min at room temperature in the dark. The cells were washed and resuspended in 0.4 mL of PBS and analyzed by using flow cytometry on a FACSCalibur cytometer (Becton-Dickinson, San Jose, CA). At least 10,000 cells were examined. The data were analyzed with Cellquest Pro software (Becton-Dickinson).

The ROS detection kit was also purchased from KeyGEN BioTECH. 2’,7’-dichlorodihydrofluorescein diacetate (H_2_DCFDA) was used to measure the amount of intracellular ROS in fresh specimens. Twenty microliters of H_2_DCFDA was added to 200 μL of cell suspension to obtain a final concentration of 10 mg/mL and incubated for 15 min at room temperature in the dark. Then, the samples were centrifuged at 300 rpm for 7 min in PBS to wash away the H_2_DCFDA. The supernatant was removed, and the remaining cell suspension was analyzed for mean fluorescence. A minimum of 10,000 cells were examined for each assay. A sample that was not stained with H_2_DCFDA or centrifuged was used as a negative control.

### Enzymatic activities

The activities of complex I, II, and III were assessed spectrophotometrically by using a commercial kit (Solarbio, Beijing, China) according to the manufacturer’s protocols, respectively.

### Determination of ATP content

ATP levels were measured in cells by using ATP determination kit (Beyotime Biotech.) according to the manufacturer’s protocol. The ATP concentration was calculated from standard curves and normalized to total protein concentration.

### RNA extraction and reverse transcription-quantitative polymerase chain reaction (RT-qPCR)

Total RNA was extracted from cells using a Total RNA Isolation Kit (BEI-BEI Biotech, Zhengzhou, China). PrimeScript RT Master Mix (Takara Bio, Inc., Otsu, Japan) was used for RT-PCR. qPCR was carried out using AceQ qPCR SYBR Green Master Mix (Vazyme Biotech, Nanjing, China) following the manufacturer’s instructions. The samples were amplified and monitored using a Roche LightCycler 96 Real-time PCR system (Roche Diagnostics, Basel, Switzerland). The thermocycling conditions were 95 °C for 10 min for initial denaturation and 40 cycles of amplification consisting of 95 °C for 10 s and 60 °C for 30 s. The fold change in gene expression was calculated using the 2^−∆∆Cq^ method with the housekeeping gene GAPDH as the internal control. The primer sequences are listed in Table S3.

### Western blot analysis

The cells were lysed in RIPA containing 1 mmol/L phenylmethylsulfonyl fluoride (PMSF) for protein extraction. Protein concentrations were analyzed using the BCA Protein Assay Kit (Thermo Fisher Scientific, Inc.), and 30 μg protein was loaded in each lane for gel electrophoresis. The procedure were performed as previously described^21^. The primary antibodies used were as follows: anti-COX-2 (1:1000), anti-CD36 (1:1000), anti-phospho-p38 (1:1000), anti-p38 (1:2000), anti-phospho-JNK (1:2000), anti-JNK (1:2000), anti-phospho-ERK1/2 (1:2000), anti-ERK1/2 (1:1000), anti-STAR (1:1000), anti-p450scc (1:1000), anti-3β-HSD (1:2000), anti-17β-HSD (1:200), anti-Ndufs1 (1:2000), anti-SdhB (1:10,000), anti-Uqcrfs1 (1:4000), anti-Fech (1:1000), anti-CytC (1:1000), and GAPDH (1:2000). The secondary antibodies used were as follows: goat anti-rabbit IgG (1:4000) and goat anti-mouse IgG (1:4000). The bands were visualized using enhanced chemiluminescence reagents (Promega Corporation, Madison, WI, USA), and images were captured using the Tanon-5200 Chemiluminescent Imaging System (Tanon Science and Technology, Co., Ltd., Shanghai, China). The relative protein expression levels were reflected by the intensities of the target bands, which were quantified using Quantity-One software.

### Human serum and semen analysis

The human study protocol was conducted by the Research Ethics Committee of Jinling Hospital and was performed in accordance with National and International guidelines. A total of 24 Chinese males (aged 24–41 years) were recruited. Males who had a history of azoospermia, cryptorchidism, varicocele, or testicular trauma who were administered hormones, or who had genital infections or other diseases during the previous 3 months were excluded. Semen samples were collected, and fasting venous blood was drawn between 8:00 a.m. and 10:00 a.m. Before initiating the study, written informed consent was obtained from all participants. The reproductive hormone and lipid levels in the human serum were measured according to routine clinical methods. The semen parameters were analyzed by the CASA system (WLJY-9000, Beijing Weili Co., Ltd., China), and sperm morphology was evaluated using the Shorr staining method. For each specimen, at least 200 spermatozoa were counted and analyzed in duplicate. The sperm DNA fragmentation index (DFI), defined as the percentage of sperm with fragmented DNA, was determined by flow cytometry using the sperm chromatin structure assay (SCSA) method.

### Statistical analysis

Data collection and analysis was performed blindly; the experimenters were unaware of the group assignment and animal treatment. All experiments were performed at least in triplicates. GraphPad Prism 6 software (GraphPad Software, Inc., La Jolla, CA, USA) was used to generate graphs. Statistical analysis was performed using SPSS 21.0 software (IBM Corp., Armonk, NY, USA). Correlations were determined by Spearman’s correlation coefficient. All data are expressed as the mean ± standard deviation(SD) which had a normal distribution. The mean and SD of the data were calculated and statistically analyzed by two-tailed Student’s *t* test and analysis of variance (ANOVA). Differences were considered to be statistically significant when *P* < 0.05 and highly significant when *P* < 0.01.

## Supplementary information

Supplementary information

Supplementary information2

Supplementary information3

Supplementary information4
