# Supplementary material for: Oxidized-LDL inhibits testosterone biosynthesis by affecting mitochondrial function and the p38 MAPK/COX-2 signaling pathway in Leydig cells
Source: Cell Death Dis. 2020 Aug 14;11(8):626. doi: 10.1038/s41419-020-02751-z (PMC7429867; doi:10.1038/s41419-020-02751-z)
Supplement: Supplementary file 1 — Supplementary information [file 41419_2020_2751_MOESM1_ESM.docx]

**Oxidized-LDL inhibits testosterone biosynthesis by affecting mitochondrial function and the p38 MAPK/****COX-2 signaling pathway in Leydig cells.**

Jun Jing^1,2†^, Ning Ding^3†^, Dandan Wang^1,2†^, Xie Ge^1,2^, Jinzhao Ma^1,2^, Rujun Ma^1,2^, Xuan Huang^1,2^, Kadiliya Jueraitetibaike^1,2^, Kuan Liang^1,2^, Shuxian Wang^1,2^, Siyuan Cao^1,2^ , Allan Zijian Zhao^3*^, Bing Yao^1,2*^

^1^ Jinling Hospital Department Reproductive Medical Center, Nanjing University, School Medical, Jiangsu, Nanjing, China.

^2^ State Key Laboratory of Reproductive Medicine, Nanjing Medicine University, Nanjing, Jiangsu, China.

^3^ The School of Biomedical and Pharmaceutical Sciences, Guangdong University of Technology, Guangzhou, Guangdong, China.

^†^These authors contributed equally to this work.

**Supplemental Figure Legends**

**Figure S1. Transfection and knockdown efficiency of siRNA-CD36 and COX-2 in TM3 Leydig cells.**

Cells were transfected with three different siRNAs targeting CD36 and COX-2, blank control or negative control (scrambled siRNA) for 24 h, and the mRNA (A, D) and protein (B, C, E, F) expression of CD36 and COX-2 was detected by qRT-PCR and Western blotting, respectively. *P < 0.05, **P < 0.01, compared with the blank control.

**Figure S2. The probable mechanism by which oxLDL negatively affects testosterone biosynthesis in Leydig cells.**
