# Supplementary figures and images for: Oxidized-LDL inhibits testosterone biosynthesis by affecting mitochondrial function and the p38 MAPK/COX-2 signaling pathway in Leydig cells
Source: Cell Death Dis. 2020 Aug 14;11(8):626. doi: 10.1038/s41419-020-02751-z (PMC7429867; doi:10.1038/s41419-020-02751-z)

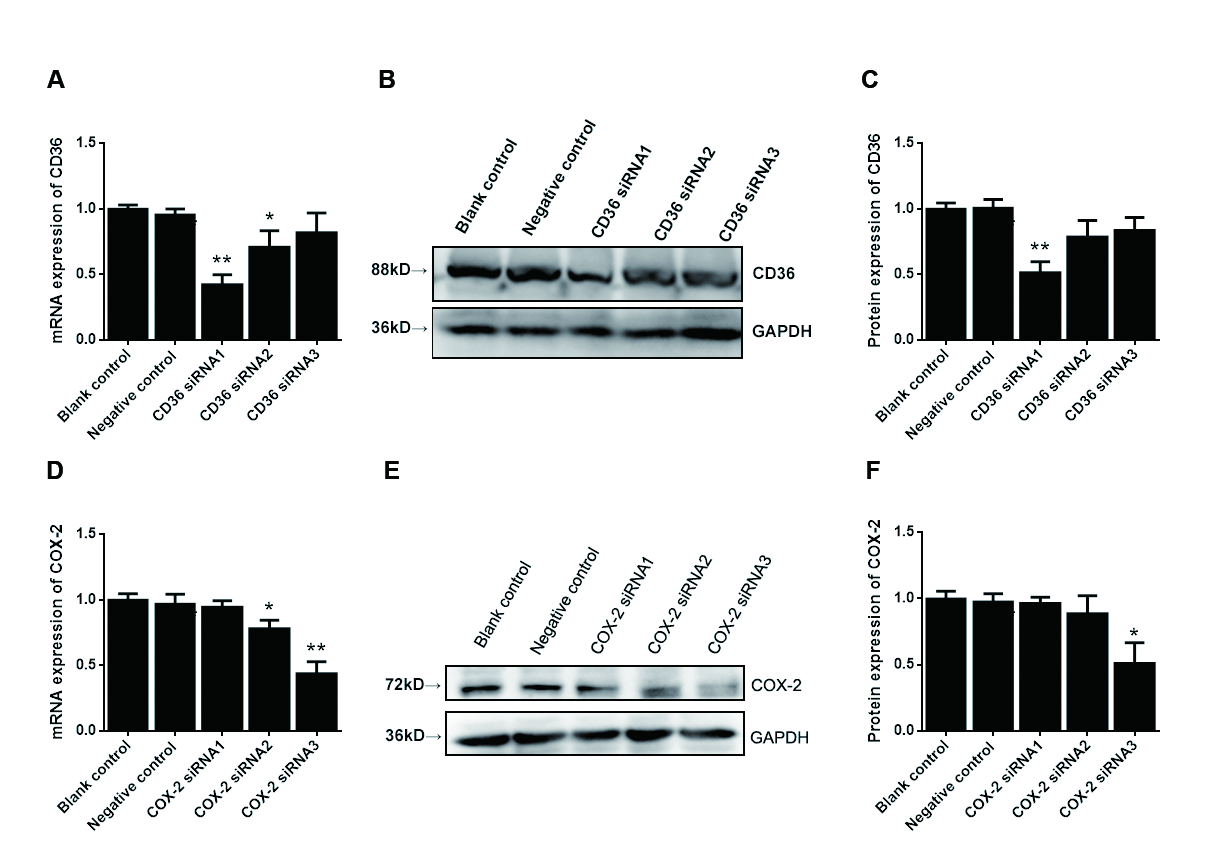

Supplement: Supplementary file 2 — Supplementary information2 [file 41419_2020_2751_MOESM2_ESM.tif]

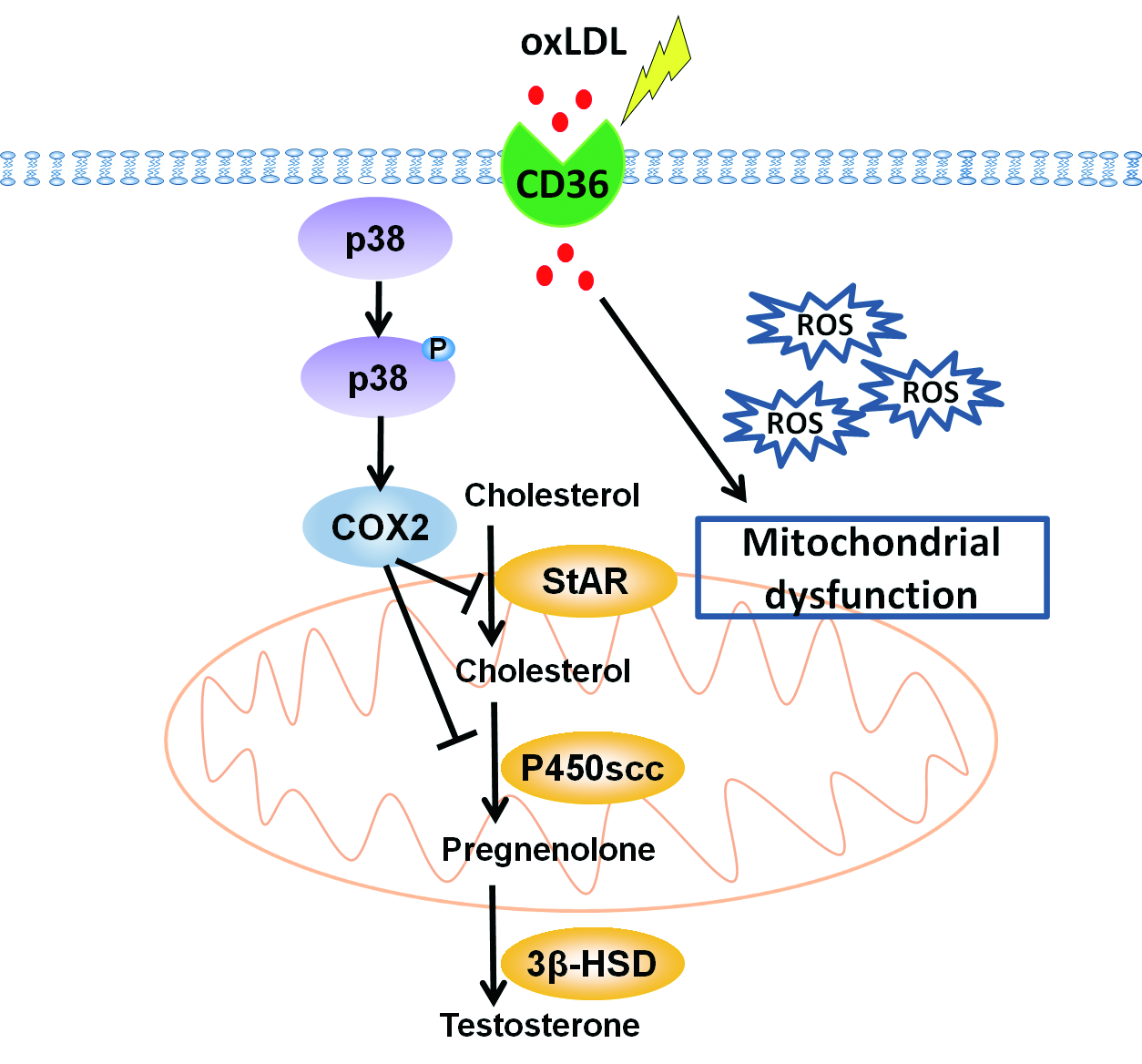

Supplement: Supplementary file 3 — Supplementary information3 [file 41419_2020_2751_MOESM3_ESM.tif]
