# Supplementary material for: Oxidized-LDL inhibits testosterone biosynthesis by affecting mitochondrial function and the p38 MAPK/COX-2 signaling pathway in Leydig cells
Source: Cell Death Dis. 2020 Aug 14;11(8):626. doi: 10.1038/s41419-020-02751-z (PMC7429867; doi:10.1038/s41419-020-02751-z)
Supplement: Supplementary file 4 — Supplementary information4 [file 41419_2020_2751_MOESM4_ESM.docx]

**Supplemental Table Legends**

**Table S1. Spearman’s correlations between the oxLDL level and sperm characteristics.**

|  | Total sperm count (×10^6^) | Sperm concentration (×10^6^/ml) | Progressive sperm motility (%) | Total sperm motility (%) | Normal sperm morphology (%) | Sperm DFI (%) |
| --- | --- | --- | --- | --- | --- | --- |
| **oxLDL** | 0.374  (0.095) | 0.384  (0.086) | 0.210  (0.360) | 0.236  (0.302) | 0.260  (0.269) | -0.128  (0.591) |

Data are presented as a correlation coefficient (*P*).

**Table S2. siRNA sequences.**

|  | siRNA sequence for CD36 (5’ to 3’) |
| --- | --- |
| CD36 siRNA1 | GGAUGACAACUUCACAGUUTT  AACUGUGAAGUUGUCAUCCTT |
| CD36 siRNA2 | CCACAUUUCCUACAUGCAATT  UUGCAUGUAGGAAAUGUGGTT |
| CD36 siRNA3 | GGAUUGGAGUGGUGAUGUUTT  AACAUCACCACUCCAAUCCTT |
|  | siRNA sequence for COX-2 (5’ to 3’) |
| COX-2 siRNA1 | UACCCGGACUGGAUUCUAUTT  AUAGAAUCCAGUCCGGGUATT |
| COX-2 siRNA2 | GCCAUGGAGUGGACUUAAATT  UUUAAGUCCACUCCAUGGCTT |
| COX-2 siRNA3 | GAGCACCAUUCUCCUUGAATT  UUCAAGGAGAAUGGUGCUCTT |

**Table S3. Primer sequences for qPCR.**

| Rat primer sequences | |
| --- | --- |
| **Gene** | Sequence (5’ to 3’) |
| StAR | Forward Primer: GACTGGAGGTGCTGCTAGAC  Reverse Primer: TACGCTTACGAAGTCTCGGG |
| P450scc | Forward Primer: GACGCCGTCTACCAGATGTT  Reverse Primer: GACACCAGGGTACTTGCTGA |
| CYP17A | Forward Primer: GTCCATAGACCTGTCCACGC  Reverse Primer: TCCACCAGATTTCTGTCGCC |
| 17β‑HSD | Forward Primer: CTCGAAGGTCTGTGCGAGAG  Reverse Primer: GCTTGCTCATAACCACGCTG |
| 3β‑HSD | Forward Primer: ATATTGGAGGCCTGCGTCG  Reverse Primer: TCGGCCATCCTTTTGCTGTA |
| GAPDH | Forward Primer: CTGCACCACCAACTGCTTAG  Reverse Primer: CCAGTGGATGCAGGGATGAT |
| Mouse primer sequences | |
| **Gene** | Sequence (5’ to 3’) |
| CD36 | Forward Primer: ATGGGCTGTGATCGGAACTG  Reverse Primer: GTCTTCCCAATAAGCATGTCTCC |
| SRA | Forward Primer: AAGTATCAGCAGAAGTCCAGTCT  Reverse Primer: TCCTTCAGTCTGAGGTCGTTG |
| LOX1 | Forward Primer: CAAGATGAAGCCTGCGAATGA  Reverse Primer: ACCTGGCGTAATTGTGTCCAC |
| LDL-R | Forward Primer: TCAGACGAACAAGGCTGTCC  Reverse Primer: CCATCTAGGCAATCTCGGTCTC |
| TLR4 | Forward Primer: GCCTTTCAGGGAATTAAGCTCC  Reverse Primer: GATCAACCGATGGACGTGTAAA |
| COX-2 | Forward Primer: TTCAACACACTCTATCACTGGC  Reverse Primer: AGAAGCGTTTGCGGTACTCAT |
| GAPDH | Forward Primer: CACCCCATTTGATGTTAGTG  Reverse Primer: CCATTTGCAGTGGCAAAG |
|  |  |
